# A proprietary alpha-amylase inhibitor from white bean (Phaseolus vulgaris): A review of clinical studies on weight loss and glycemic control

**DOI:** 10.1186/1475-2891-10-24

**Published:** 2011-03-17

**Authors:** Marilyn L Barrett, Jay K Udani

**Affiliations:** 1 Pharmacognosy Consulting, Mill Valley, CA 94941, USA; 2 Medicus Research LLC, Northridge, CA 91325, USA; 3 UCLA School of Medicine, Department of Medicine, Los Angeles, CA 90024, USA

## Abstract

Obesity, and resultant health hazards which include diabetes, cardiovascular disease and metabolic syndrome, are worldwide medical problems. Control of diet and exercise are cornerstones of the management of excess weight. Foods with a low glycemic index may reduce the risk of diabetes and heart disease as well as their complications. As an alternative to a low glycemic index diet, there is a growing body of research into products that slow the absorption of carbohydrates through the inhibition of enzymes responsible for their digestion. These products include alpha-amylase and glucosidase inhibitors. The common white bean (Phaseolus vulgaris) produces an alpha-amylase inhibitor, which has been characterized and tested in numerous clinical studies. A specific and proprietary product named Phase 2^® ^Carb Controller (Pharmachem Laboratories, Kearny, NJ) has demonstrated the ability to cause weight loss with doses of 500 to 3000 mg per day, in either a single dose or in divided doses. Clinical studies also show that Phase 2 has the ability to reduce the post-prandial spike in blood glucose levels. Experiments conducted incorporating Phase 2 into food and beverage products have found that it can be integrated into various products without losing activity or altering the appearance, texture or taste of the food. There have been no serious side effects reported following consumption of Phase 2. Gastro-intestinal side effects are rare and diminish upon extended use of the product. In summary, Phase 2 has the potential to induce weight loss and reduce spikes in blood sugar caused by carbohydrates through its alpha-amylase inhibiting activity.

## Review

Obesity is a major health hazard, with increased risk for cardiovascular disease (mainly heart disease and stroke), type 2 diabetes, musculoskeletal disorders (especially osteoarthritis) and certain types of cancer (endometrial, breast, and colon) [1]. The World Health Organization (WHO) estimated that in 2005, approximately 1.6 billion adults worldwide were overweight and at least 400 million were obese. Further, the WHO estimated that at least 20 million children under the age of 5 years were overweight. The projected numbers for 2015 are larger, with 2.3 billion adults expected to be overweight and 700 million expected to be obese [1].

The cause of excess body weight is an imbalance between energy intake and expenditure. The WHO has identified a global shift in diet towards increased intake of energy-dense foods that are high in fat and sugars but low in vitamins, minerals and other micronutrients. At the same time there is a trend towards decreased physical activity due to the increasingly sedentary nature of many forms of work, changing modes of transportation, and increasing urbanization [1].

Control of diet and exercise are cornerstones of the management of excess weight. A number of nutritional approaches and diets with difference proportions of lipids, proteins and carbohydrates have been prescribed for weight loss. Initial guidance on weight loss was a restriction in saturated fats. However diets low in saturated fats did not necessarily result in weight loss as expected. More recently there has been a shift towards a reduction in carbohydrates, particularly refined carbohydrates, as an approach to reduce weight and the incidence or related disease risk [2].

In most diets, carbohydrates are the greatest source of calories. Carbohydrates are polyhydroxy aldehydes, ketones, alcohols and acids that range in size from single monomeric units (monosaccharides) to polymers (polysaccharides). Before being absorbed by the body, carbohydrates must be broken down into monosaccharides. This breakdown occurs due to two major enzymes: amylase and glucosidase [3].

Digestion of carbohydrates begins in the mouth, with amylase secreted by salivary glands. This action accounts for only about 5% of the breakdown of carbohydrates. The process is halted in the stomach due to the high acid environment destroying the amylase activity. When the food enters the intestine, the acidic pH is neutralized by the release of bicarbonate by the pancreas and by the mucous that lines the walls of the intestine. Amylase is secreted into the small intestines by the pancreas. Alpha-glucosidase enzymes are located in the brush border of the small intestines. Amylase breaks down the carbohydrates into oligosaccharides. The glucosidase enzymes (including lactase, maltase and sucrose) complete the breakdown to monosaccharide units. It is only the monosaccharide units that are absorbed into the body. Glucose and other monosaccharides are transported via the hepatic portal vein to the liver. Monosaccharides not immediately utilized for energy are stored as glycogen in the liver or as fat (triglycerides) in adipose tissue, liver and plasma. Carbohydrates that are resistant to digestion in the intestine enter the colon, where they are fermented by colonic bacteria to produce short-chain fatty acids, carbon dioxide and methane.

Dietary carbohydrates that are composed mostly of monosaccharide units are absorbed quickly and are said to have a "high glycemic index". Carbohydrates in polymeric form are absorbed more slowly and said to have a "low glycemic index". The glycemic index (GI) is defined as the incremental area under the blood glucose curve following ingestion of a test food, expressed as a percentage of the corresponding area following an equivalent load of a reference carbohydrate, either glucose or white (wheat) bread [4]. Factors that influence the GI besides the composition of the carbohydrate are the fat and protein content of the food, the acidity of the food and the presence of fiber [5]. Low GI foods (< 55) include vegetables, unsweetened yogurt and protein-enriched spaghetti. High GI foods (> 70) include white bread, baked potato and dates.

After consumption of high GI foods, there is a large, rapid increase in blood sugar levels and in response a rapid increase in insulin levels. Insulin promotes the uptake of glucose from the blood into cells in the liver and skeletal muscle tissue, storing it as glycogen. Insulin also increases fatty acid synthesis and can result in the accumulation of lipids. Accumulation of lipids in skeletal muscle and the liver is associated with a decrease in insulin sensitivity. Insulin resistance increases the chance of developing type-2 diabetes and heart disease. Post-prandial hyperglycemia and insulin resistance are thought to play a central role in the development and progression of cardiovascular disease in subjects with impaired glucose tolerance. Post-prandial hyperglycemia is associated with endothelial dysfunction and an increase in intima-media thickness as well as a higher prevalence of atherosclerotic plaques. High glucose levels have been shown to stimulate expression of adhesion molecules (intercellular adhesion molecule-1, vascular adhesion molecule-1, E-selectin) and cytokines in in-vitro models. Hyperglycemia causes an increase in oxidative stress with associated oxidation of low-density lipoprotein, platelet activation and thrombin generation [5,6]. A body of evidence, including prospective cohort studies, randomized controlled trials and mechanistic experiments, support a role for low GI diets in the prevention of obesity, diabetes and cardiovascular disease [7-9]. Three large-scale epidemiological studies on women reported a correlation between a high glycemic index diet and the incidence of type 2 diabetes [10-12]. The populations studied were 59,000 US black women, 65,000 Chinese women and 91,249 US nurses, who were each followed for periods of time of 5 to 8 years. Another prospective cohort study in Europe, which included 25,000 men and women, concluded that high cereal fiber was inversely associated with the risk of developing diabetes [13].

As previously indicated, the choice of the type of carbohydrate foods in the diet, with their varying glycemic properties, with determine the rate of absorption of sugars into the body. One means of reducing the GI of a meal is the inclusion of resistant starches. Resistant starches are those that resist digestion in the small intestine, thereby passing into the large intestine, where they act like dietary fiber [14]. These starches are naturally found in seeds, legumes and unprocessed whole grains. The amount of resistant starch in food is influenced by processing, which can either increase or decrease the amounts found in the raw substance. Resistant starch can be added to foods such as bread, biscuits, sweet goods, pasta, nutritional bars and cereal, in order to lower their GI index, without affecting taste or texture [15,16].

An alternative to a low GI diet are products that slow the absorption of carbohydrates through the inhibition of enzymes responsible for their digestion. These products include alpha-amylase and glucosidase inhibitors. Acarbose (Prandase^®^, Precose^®^) is a prescription drug, which inhibits alpha-glucosidase enzymes in the brush border of the small intestines and pancreatic alpha-amylase. Other drugs that belong to this class are miglitol and voglibose. Acarbose reduces post-prandial hyperglcemia and is used to treat diabetes type-2. Clinical studies with subjects with impaired glucose tolerance have demonstrated not only an improvement in post-prandial hyperglycemia but also cardiovascular benefits. Acarbose has been shown to slow the progression of thickening of the intima-media in the carotid arteries, reduce the incidence of cardiovascular disease and reverse newly diagnosed hypertension. Recently acarbose has been reported to improve insulin resistance in subjects with impaired glucose tolerance or diabetes type-2. Due to these findings, acarbose has been suggested as treatment to reduce cardiovascular risk in subjects with metabolic syndrome (a cluster of risk factors including high triglycerides, low high-density lipoprotein cholesterol and hypertension) [6].

Alpha-amylase inhibitors with activity against mammalian forms of the enzyme are present in plants and it is suggested that they were developed by plants in order to strengthen their defense against predators. Plant constituents with enzymatic inhibitory activity include polyphenolic compounds and glycoproteins [17]. For example, anthocyanins and ellagitannins present in raspberries and strawberries have been reported to inhibit alpha-glucosidase and alpha-amylase activity, respectively [18]. In addition, theaflavins and catechins present in green and black teas have been reported to inhibit alpha-amylase and alpha-glucosidase activity as well as retard starch digestion in an in-vitro model [19]. Alpha-amylase inhibitors are also present in grains, including wheat and rice [17]. However, the greatest body of research has gone into glycoproteins extracted from kidney beans (Phaseolus vulgaris) and more specifically on the proprietary Phase 2 product.

### Properties of alpha-amylase inhibitors from beans

Common beans have 3 isoforms of alpha amylase inhibitor (alpha-A1, alpha-A12, alpha-AIL). The alpha-AI isoform has anti-amylase activity in humans. This enzyme is found in the embryonic axes and cotyledons in the seed and not in other organs of the plant. It is not active against plant alpha-amylases and is therefore classified as an anti-feedant or seed defense protein [20].

The alpha amylase inhibitor prevents starch digestion by completely blocking access to the active site of the alpha-amylase enzyme. Factors that affect the activity of the alpha-AI isoform inhibitor are pH, temperature, incubation time and the presence of particular ions. The optimum pH for the inhibitor is 4.5 to 5.5 and the optimal temperature is 22 to 37°C. There is no activity at 0°C and the inhibitor is completely inactivated by boiling for 10 minutes. The ideal incubation period has been recorded as 10 minutes, 40 minutes and 120 minutes by three different researchers [3]. The different incubation times are thought to be due to the use of different test conditions; namely a pH of 6.9 for the longer incubation periods and a pH of 4.5 for the shortest [3].

## Background Experiments in Humans

In the early 1980's several products containing crude preparations of bean amylase inhibitors were marketed in the United States. However early clinical studies were disappointing and it was discovered that the preparations had insufficient enzyme inhibiting activity, as well as issues with potency and stability. Subsequently, a research group at the Mayo Clinic developed a partially purified white bean product and published a series of studies exploring the activity of inhibitor in human clinical studies. The test product was described as a concentrate: 6 to 8-fold by total protein content and 30 to 40 fold by dry weight [21]. The product was found to inactivate salivary, intraduodenal and intraileal amylase activity in vitro. Its activity was not affected by exposure to gastric juice and only minimally by duodenal juice (by 15%). In vitro studies demonstrated that the inhibitor decreased digestion of dietary starch in a dose-dependent manner [21]. Perfusion of the white bean product into the duodenum of human subjects completely inhibited the activity of intraluminal amylase activity (5.0 mg/ml at 5 ml/min) [21]. Subsequent experiments were conducted with volunteers intubated with an oroileal tube in order to obtain duodenal, jejuna and terminal ileal samples [22]. After intubation the subjects ingested 50 g rice starch and on the subsequent day they ingested starch with the amylase inhibitor (5 g or 10 g white bean extract). The white bean extract significantly reduced duodenal, jejunal, and ileal intraluminal amylase activity by more than 95%; it acted as quickly as 15 minutes, and for as long as 2 hours. It increased the post prandial delivery of carbohydrates to the distal small bowel by 22 to 24% (as measured by oroileal tube aspiration) and increased hydrogen concentrations in the breath from 30 to 90 minutes after the meal. Hydrogen breath testing is an accepted method of determining carbohydrate malabsorption as colonic bacteria ferment carbohydrates into organic acids, carbon dioxide and hydrogen. A percentage of these gases are absorbed into the portal blood stream and subsequently expired through the lungs [23-25]. The white bean extract also reduced the postprandial plasma glucose rose by 85% and eliminated the subsequent fall of glucose level to below fasting levels. The extract significantly lowered the postprandial plasma levels of insulin, C-peptide and gastric inhibitory polypeptide [22].

A follow-up study using subjects with diabetes mellitus demonstrated a decrease in the postprandial increases in plasma glucose and insulin levels [26]. Further studies revealed that a dose of 3.8 g white bean inhibitor could cause more than twice the amount of hydrogen in the breath following a standard spaghetti meal. The percentage of malabsorbed carbohydrate increased from 4.7% to 7.0% (p < 0.05). Also, the form of the inhibitor (powder, tablet) had no effect on the activity when taken with the spaghetti [27]. Follow-up studies found that a dose of 2.9 g was sufficient to significantly inhibit the postprandial increases in blood glucose, C-peptide and gastric inhibitory polypeptide following 650-calorie meal containing carbohydrate, fat and protein [28]. A longer-term study was conducted over 3 weeks with 6 non-insulin dependent diabetics. The subjects were given sufficient white bean inhibitor to reduce the increase in postprandial plasma glucose by more than 30%: a dose of 4 to 6 g with each meal. As a result there were significant decreases in postprandial glucose, C-peptide, insulin and gastric inhibitory polypeptide along with a significant increase in hydrogen excretion in the breath. Diarrhea and gastrointestinal symptoms occurred the first day of administration of the inhibitor and resolved over the next couple of days [29]. A further experiment with 18 healthy subjects reported that carbohydrates perfused into the ileum delayed emptying of a meal infused into the stomach. In one half of the subjects, the amylase inhibitor was added to the ileum perfusate. The inhibitor significantly reduced the absorption of carbohydrate from the ileum and enhanced the delay in gastric emptying. Plasma concentrations of C-peptide, glucagon, motilin, gastrin and human pancreatic polypeptide were not influenced by changes in gastric emptying or by the ileal perfusates. However the delay in gastric emptying was significantly associated with a decrease in plasma concentrations of gastric inhibitory polypeptide and neurotensin along with an increase in concentrations of peptide YY. This effect was caused by the delay in gastric emptying, rather than the other way around. Human polypeptide levels were not changed and the authors concluded that the hormonal changes were not mediated via the vagus nerve [30].

### Phase 2 specifications

The Phase 2^® ^product is a water extract of the white kidney bean (Phaseolus vulgaris) standardized to alpha-amylase (8;12;15;39) inhibiting units (Pharmachem Laboratories, Kearny, NJ). Phase 2 is produced from non-GMO whole white kidney beans, which are ground and then extracted for 4 hours. The liquid is filtered and concentrated under vacuum. The extract is filtered again, and then pasteurized before being spray dried. Phase 2 is odorless and tasteless. Each lot of Phase 2 has at least 3000 alpha amylase inhibiting units (AAIU) per g when tested at a pH 6.8 using potato starch as the substrate and pancreatin as the enzyme source. The Phase 2 extraction process was designed to make it more potent and stable than the white bean product tested by the Mayo clinic.

Phase 2 is used as a dietary supplement in various forms, including powders, tablets, capsules and chewables. There are approximately 200 brands of nutritional supplement/weight loss products in the worldwide market that contain Phase 2. A typical dose is 1 to 2 capsules, each containing 500 mg, taken before each of 3 daily meals, for a total of 1500 to 3000 mg per day. A private safety panel approved a maximum daily intake of 10,000 mg (10 g) [31].

Experiments conducted incorporating Phase 2 into food products have found that it can be incorporated into chewing gum, mashed potatoes, yeast-raised dough (bread, pizza, etc) without losing activity or altering the appearance, texture or taste of the food [32-34].

### Clinical studies conducted with Phase 2

Ten clinical studies have demonstrated weight loss over time following administration of Phase 2. Three studies demonstrated significant loss of body weight with Phase 2 compared to a placebo control in people who are overweight or obese. The doses ranged from 445 mg for 4 weeks to 3000 mg for 8 to 12 weeks [35-37]. A placebo controlled study by showed a comparative loss in body weight only when subjects were stratified by dietary carbohydrate intake. Those who consumed the greatest amount of carbohydrate, lost significant body weight in comparison to the placebo group [38]. Six additional studies reported a loss of weight over time [39-44]. Three clinical studies reported a reduction in serum triglycerides over time [40,41,44] (Table 1).

**Table 1:** Phase 2 Clinical Data

Reference (Author, date)	Study Design/Duration	Subjects	Purpose	Preparation/Dose	Main Results
Thom, 2000 [39]	RPCT, 12 weeks	n = 40 (BMI 28-39)	weight loss	400 mg Phaseolamin^® ^3X after meals, total 1200 mg/day (other ingred. inulin &*Garcinia *extract)	↓body weight, BMI & %body fat in active group (p < 0.05), no effect in placebo group; no between group analysis

Erner, 2003 [40]	RPCT, 12 weeks, then open 12 weeks	n = 54 (BMI 24-36)	weight loss	Thera-Slim: 1000 mg Phase2 before 2 meals, total 2000 mg/day	trend toward ↓body weight, 3X decrease in triglycerides; no between group analysis

Rothacker, 2003 [36]	RPCT, 12 weeks	n = 88 (BMI 24-32)	weight loss	StarchAway chews: 1000 mg Phase2 before 3 meals, total 3000 mg/day	↓body weight comparison to placebo (p < 0.05)

Udani, 2004 [44]	RPCT, 8 weeks	n = 39 (BMI 30-43)	weight loss	Phase 2 1500 mg 2X, 3000 mg/day	↓body weight comparison to placebo (ns) ↓trigylcerides (ns)

Koike, 2005 [41]	Open, 8 weeks	n = 10 (BMI 23-30)	weight loss	3 capsules Phaseolamin 1600 diet 2X daily; 750 mg Phase 2 daily	↓body weight (p = 0.002), calorie intake, BMI, triglycerides & HDL (all p < 0.05)

Osorio, 2005 [42]	Open, 30 days	n = 39 (overweight & obese)	Weight loss	PreCarb capsules: 1000 mg Phase3 with meals, total 3000 mg/day	↓body weight & ↓waist to hip ratio over time (both p < 0.001)

Celleno, 2007 [35]	RPCT, 30 days	n = 60 (BMI avg 26)	Weight loss	Phase 2 + chromium; 445 mg extract daily	↓body weight (p < 0.001), BMI, body fat (both p < 0.01)

Vinson, 2009 [45]	PCT, X-over, single dose	Part 1: n = 11, Part 2: n = 7	Plasma glucose	Phase 2 mixed with margarine or gravy. 750 or 1500 mg.	↓AUC post prandial blood glucose; higher dose (p < 0.05).

Udani, 2009 [46]	RPC, X-over, single dose	n = 13 (BMI 18-25)	Plasma glucose	Phase 2 capsules or mixed w/butter. 1500, 2000, 3000 mg	↓AUC post prandial blood glucose; 3000 mg w/butter (p < 0.05).

Wu, 2010 [37]	RPCT, 60 days	n = 101 (BMI 25-40)	Weight loss	Phase 2; 1,000 mg 3X daily	↓body weight, waist circumference (both p < 0.01)

DB = double-blind, PCT = placebo-controlled trial, RPCT = randomized placebo-controlled trial, X-over = crossover

### Weight loss - compared to placebo

A 12 week randomized double-blind placebo controlled trial included 60 overweight individuals (BMI between 24 and 32 kg/m^2^). The subjects consumed 2 soft chews before each meal containing either Phase 2 (500 each mg) or placebo for 12 weeks. The Phase 2 group consumed a total of 3000 mg Phase 2 per day. A total of 88 men and women enrolled in the study, while 60 completed the study and were included in the analyses. There was a statistically significant weight reduction in the active group compared with the placebo group at weeks 6, 8 and 12. The amount of weight lost by the active group at 12 weeks was 6.9 ±7.9 lbs (average of 0.575 lbs per week) while the placebo group gained 0.8 lbs ±6.1 (p = 0.029 between groups). There were no significant differences between groups in body fat, lean body mass or body measurements (waist/hip circumferences). No adverse events were reported [36].

A randomized, double-blind, placebo-controlled study was conducted with 60 slightly overweight subjects (5 to 15 kg overweight). The subjects were required to have a stable weight for the past 6 months and underwent a 2-week single-blinded, run-in period prior to randomization. The subjects took 1 tablet (active or placebo) per day for 30 consecutive days before a meal rich in carbohydrates (2000 to 2200 calorie diet). The active tablet contained 445 mg Phase 2 and 0.5 mg chromium picolinate (≈55 mcg elemental chromium). After 30 days, the active group had a significant reduction in body weight, BMI, fat mass, adipose tissue thickness and waist/hip/thigh circumferences while maintaining lean body mass. The active group lost an average of 2.93 kg (6.45 lbs) in 30 days compared with an average of 0.35 kg (0.77 lbs) in the placebo group (p < 0.001). BMI in the test group was reduced from an initial 25.9 ± 2.0 (SEM) to 24.9 ± 1.9 (p < 0.01). The placebo showed no significant change from the initial 26.0 ± 2.3 (SEM). Body composition was measured with bioelectrical impedance. The active group demonstrated a 10.45% reduction in body fat compared with a 0.16% reduction in the placebo group (p < 0.001). Waist and hip circumferences measured in a standard way, showed the same pattern as well. The active group demonstrated 2.93 cm and 1.48 cm reductions respectively, compared with 0.46 cm and 0.11 cm reductions in the placebo group (p < 0.001). No adverse events were reported [35].

A randomized, double-blind, placebo-controlled study was conducted in China with 101 volunteers who had a BMI between 25 and 40. The subjects were given a single capsule containing 1000 mg Phase 2 or placebo three times per day, just before meals, for 60 days. The active group ingested a total of 3,000 mg Phase 2 per day. As a result, there was significant weight loss in the active groups compared to the placebo group after 30 and 60 days. After 60 days, the average weight loss in the active group was 1.9 ± 0.15 kg compared to 0.4 ± 0.13 kg in the placebo group (p < 0.001). There was also a significant reduction in waist measurement in the active group compared to the placebo group (1.9 ± 0.32 cm compared to 0.4 ± 0.26 (p < 0.001). There was no effect on hip measurements. Blood chemistries did not change significantly over the 2 month study and no adverse side effects were reported [37].

A 4-week, randomized, double-blind, placebo-controlled study conducted with 25 healthy overweight (BMI 25-30) subjects [38]. The subjects took 1000 mg of Phase 2^® ^or an identical placebo twice a day (before breakfast and lunch) as part of a weight loss program which included diet, exercise and behavioral intervention. The subjects were given nutritional guidelines to standardize their caloric intake at 1800 Kcal/day. Breakfast and lunch were provided to increase compliance. In addition, subjects met with a personal trainer to establish an exercise program and had a counseling session with a behavioral psychologist to identify psychological barriers to weight loss. As a result of this intervention, both groups reduced weight and waist size significantly compared to baseline, but there were no significant differences between groups. After 4 weeks, the active group lost 6.0 lbs and the placebo group lost 4.7 lbs compared to baseline (p = 0.0002 active and p = 0.0016 placebo). The active group lost a mean of 2.2 in inches from their waists and the control group lost 2.1 inches from their waists compared to baseline (p = 0.050 and 0.0001, respectively). For exploratory analysis, subjects were stratified by dietary carbohydrate intake. In this analysis, the tertile that took in the most carbohydrates demonstrated significantly greater loss of body weight compared with the placebo group (8.7 pounds vs. 1.7 pounds, p = 0.04). This group also had a significantly greater loss in inches around the waist (3.3 vs 1.3 inches; p = 0.01). There were no significant changes from baseline in hip circumference, triglycerides, fasting glucose, total cholesterol, appetite control, hunger, energy level, and percent body fat, neither were there any significantly differences between groups. No side effects or adverse events were reported [38].

### Weight loss - over time

In a randomized double blind placebo controlled trial, forty healthy overweight (BMI 27.5 to 39.0) were randomized and instructed to take 2 tablets of the test product immediately after all 3 meals (breakfast, lunch and dinner) for 12 weeks [39]. Subjects were also instructed to follow a 1200 kcal/day low-fat diet. The tablets, 650 mg each, contained a proprietary blend (Suco-Bloc^®^) including 200 mg of Phase 2 (Phaseolamin^®^, Leuven Bioproducts, Belgium), 200 mg of inulin (from chicory root), and 50 mg of Garcinia cambogia extract. The remaining 200 mg in the tablets were not described. All subjects were included in an intent-to-treat analysis, including 7 subjects who dropped out of the study (6 in the placebo arm, 1 in the active arm). After 12 weeks, the active group had a significant reduction in weight, BMI and percent body fat compared to baseline, whereas there was no significant change in the placebo group. The active group lost an average of 3.5 kg (7.7 lb; p = 0.001) and the placebo group lost 1.3 kg (2.9 lb). BMI decreased by 1.3 kg/m^2 ^(p = 0.01) in the active group and by 0.5 kg/m^2 ^in the placebo group. Percent body fat (measured by bioelectrical impedance) decreased by 2.3% (p = 0.01) in the active group and by 0.7% in the placebo. Body mass analyses showed that the weight loss in the active group consisted mainly of fat loss as >85% of the weight loss was accounted for by fat. Between group analyses was not provided for any of the variables. No adverse events were reported in either group [39].

A 12 week double-blind, placebo-controlled study was conducted and this period was followed by an additional 12 weeks were in all the participants received the active treatment [40]. In the first part of the study, the subjects took 2 capsules twice a day of placebo or Thera-Slim™. Thera-Slim™ capsules contained 500 mg Phase 2 plus 250 mg fennel seed powder. The placebo contained cellulose and fennel seed powder. The active group received a total of 2000 mg Phase 2 per day. The subjects were asked to eat a diet in which lunch and dinner contained 100 to 200 g of carbohydrates. Sixty overweight and obese adult subjects (BMI 24-36) were randomized and 54 completed the study. After the first 12 weeks, the active group lost a average of 1.4 lbs and the placebo gained an average of 0.6 lbs. Serum triglyceride levels dropped by almost 3.3 times in the active group compared to the placebo group (-38.1 vs -11.9). The levels of total cholesterol and HDL were similar in both groups. No between group analyses were included in the report. There were no adverse events reported after 24 weeks of usage.

A randomized double blind placebo controlled study was conducted with 39 obese subjects (BMI 30-43) who were randomly allocated to receive either 1500 mg of Phase 2 or an identical placebo twice daily with lunch and dinner for 8 weeks [44]. The active group received a total of 3000 mg Phase 2 per day. Subjects were instructed to consume a controlled high fiber/low fat diet that provided 100 to 200 g of complex carbohydrate intake per day. Subjects were also instructed to eat the majority of their carbohydrates during lunch and dinner since those were the meals at which the Phase 2 or placebo were taken. The amount of carbohydrate intake was determined for the subjects on the basis of their estimated daily maintenance carbohydrate requirement. Twenty seven subjects completed the study (14 active and 13 placebo). After 8 weeks the active group lost an average of 3.79 lbs. (an average of 0.47 lbs. per week) compared with the placebo group which lost an average of 1.65 lbs. (an average of 0.21 lbs. per week). The difference was not statistically significant with a two tailed p-value of 0.35. Triglyceride levels in the Phase 2 group were reduced by an average of 26.3 mg/dl. This reduction was more than three times the average reduction of 8.2 mg/dl seen in the placebo group (p = 0.07).

Several secondary outcomes were measured during the study including body fat percentage, waist and hip circumferences, energy level, hunger, appetite, HbA1c, and total cholesterol. For each of these secondary measures, no clinically or statistically significant differences were identified between the active and the placebo group. No adverse events occurred that were felt to be due to the active product. One placebo subject experienced abdominal pain, bloating and gas while one active group subject complained of an increased incidence of tension headaches. There were no clinically significant changes in biochemical indicators of safety, including serum electrolytes, and markers of kidney and liver function [44].

### Weight loss - open studies

An open study was conducted with 10 healthy subjects (5 men and 5 women) with a BMI between 23 and 30 and a body fat ratio of over 25% for men and over 30% for women [41]. The subjects took 3 capsules of Phaseolamin™ 1600 diet twice a day, 30 min before lunch and dinner, for 8 weeks. The six capsules (1.5 g) contained 750 mg Phase 2, 200 mg clove, 20 mg lysine, 20 mg arginine, 20 mg alanine.

Over the course of 8 weeks, caloric intake decreased from 1742 ± 254 kcal/day to 1525 ± 249 kcal/day (p = 0.01) and the subjects lost a significant amount of weight (2.4%; 74.5 ± 7.3 to 72.7 ± 7.8; p = 0.002). There were also a significant reduction in body fat (p < 0.001) and BMI (p = 0.002). There were reductions in waist and of hip circumferences, without a significant change in the ratio of waist to hip circumference. Over the 8 weeks there were significant reductions in systolic and diastolic blood pressure (p = 0.01 and p < 0.001). There were also significant reductions in triglycerides (p = 0.019) and HDL cholesterol (p = 0.001), but not in total cholesterol or LDL cholesterol. There was no change in blood glucose levels and no adverse events were reported [41].

An open study was conducted with 50 healthy adult subjects who were overweight or obese. They were given Precarb (Natrol's Carb Intercept 500 mg capsules) containing Phaseolamin/Phase2 [42]. The subject took 1 g Precarb (2 capsules, 3 times daily with high carbohydrate meals) for 30 days. A per-protocol analysis was conducted on the 37 to 39 subjects who completed the study. There was a significant reduction in mean body weight of 2.34 ± 2.21 kg (n = 37; p < 0.001) and a significant reduction in mean waist-to-hip ratio 2.77 ± 2.55 (n = 39; p < 0.001).

In an open label study 23 adult men and women (BMI 22) took "Super Bows Diet Type B", a granular food available in Japan that contains 500 mg Phase 2, Coleus forskohlii extract and mushroom chitosan (Plus fort Barrious^®^) for a period of 8 weeks [43]. Bows Diet Type B was taken as 1 packet of powder in a glass of water 20 minutes before lunch and dinner. After 8 weeks, the product caused a significant decrease in body weight (0.78 ± 0.20 kg, p < 0.01) and percent body fat (1.19 ± 0.37%, p < 0.01). There was no change on calorie intake during this period. In 10 subjects who had a BMI over 24 and a total cholesterol over 220 mg/dl, there was a significant decrease in cholesterol after 4 and 8 weeks (25.3 ± 7.1 mg/dl and 11.3 ± 4.0 mg/dl, respectively; both p < 0.05). There were temporary gastrointestinal symptoms such as bloating and constipation but these symptoms disappeared following a few days of continuous intake of the product.

### Glycemic Index (GI)

Four cross-over clinical studies addressed the potential effect of Phase 2 on post-prandial increases in blood sugar. All four studies indicated that Phase 2 could reduce post-prandial spikes in blood sugar with a suggestion that the effect is dose-related.

In the first study, a placebo-controlled, cross-over study, eleven fasting subjects (men and women aged 21 to 57) were given 4 slices of white bread and 42 g (3 Tbs) of margarine with or without 1500 mg of Phase 2 (the Phase 2 was added to the margarine) [45]. The food contained a total of 610 calories, 60.5 of which came from carbohydrate. The tests were administered a week apart. Absorption and metabolism of carbohydrate was measured as levels of plasma glucose over time. In comparison to control, the glucose levels following consumption of Phase 2 returned to baseline 20 minutes earlier. The area under the plasma glucose vs. time curve was 66% lower with Phase 2 compared to the control (p < 0.05). The authors concluded that this indicated that 1/3^rd ^of the carbohydrate in the bread was absorbed. However, actual absorption and subsequent excretion was not measured.

The second study, published in the same paper, was also a placebo-controlled, cross-over study. Seven subjects (men and women 23 to 43 years old) were given a frozen dinner containing country fried steak, mash potatoes, green beans and cherry-apple pie (630 calories with 64 g carbohydrate) with and without 750 mg Phase 2. In this study, the Phase 2 was mixed with the gravy. The effect of Phase 2 was to reduce the average plasma glucose vs. time curve by 28% and the authors concluded that 2/3rds of the carbohydrate in the meal was absorbed. The authors noted that there appeared to be a dose-related effect with the 1500 mg dose of Phase 2 being twice as effective as the 750 mg dose [45].

A 6-arm crossover study was conducted with 13 randomized subjects (BMI 18-25) to determine whether the addition of Phase 2 would lower the GI of a commercially available high glycemic food (white bread) [46]. Standardized GI testing was performed using capillary blood glucose measurements following ingestions of white bread with butter, with and without the addition of Phase 2 in capsule or powder form. In both formulations, Phase 2 was given in dosages of 1500 mg, 2000 mg, and 3000 mg. The powdered form was mixed with the butter. Statistical analysis was performed by one-way ANOVA of all seven treatment groups using unadjusted multiple comparisons (t tests) to the white bread control. For the capsule formulation, the 1500 mg dose had no effect on the GI and the 2000 mg and 3000 mg capsule doses caused insignificant reductions in GI. For the powder, the 1500 mg and 2000 mg doses caused insignificant reductions in the GI, while the 3000 mg dose caused a significant reduction in post-prandial glucose levels (a reduction of 34.11%, p = 0.023).

A single dose double-blind cross-over test was conducted on the effects of Super Bows Diet Type B on blood sugar levels [43]. As previously stated, this product is a granular food available in Japan that contains 500 mg Phase 2, Coleus forskohlii extract and mushroom chitosan. The experiment included 13 men and women with a fasting blood glucose level above 126 mg/dl. In two test periods 1 week apart, the subjects took a packet of product or placebo along with a glass of water 5 minutes before eating 300 g polished rice. Blood samples were taken before the intake of the rice and 30, 60, 90 and 120 minutes afterward. Blood sugar levels 30 minutes after eating the rice were significantly lower with the test product (p < 0.01). Plasma insulin levels were significantly lower compared to the control at 30 and 60 minutes after consuming the rice (p < 0.01).

### Safety

In the human clinical studies reviewed above there were no reports of serious side effects resulting from ingestion of white bean extracts. Clinical efficacy studies using doses of Phase 2 up to 3000 mg per day in divided doses for periods of 30 days to 24 weeks also reported no significant adverse events. An acute animal toxicity study was conducted in rats with Phase 2 at doses of 500 to 5000 mg/kg body weight along with a subchronic study of 90 days with doses of 200 to 1000 mg/kg. In response, there were no adverse reactions and signs of toxicity in biochemical and histopathological analysis [47]. A 28-day toxicity study conducted with male and female rats reported a no-adverse-effect level (NOAEL) of 2500 mg/kg/day [48]. Cantox Health Sciences International conducted a safety review of published and unpublished data on Phase 2 and the panel of experts concluded that it could be safety consumed at doses up to 10 g per day [31].

Since alpha-amylase inhibitors prevent the degradation of complex carbohydrates into oligosaccharies, those carbohydrates will pass through the intestine into the colon. In the colon, bacteria will digest the complex carbohydrates, and this may initially cause gastrointestinal side-effects such as flatulence and diarrhea. In the study conducted with Super Bows Diet Type B which contained Phase 2 along with other ingredients, there were temporary gastrointestinal symptoms including bloating and constipation but these symptoms resolved with continued intake of the product [43].

Raw beans contain phytohaemagglutinin (PHA) at high levels which have been associated with toxic effects in animals and severe gastrointestinal disturbances in humans [3]. However, PHA levels in beans are drastically reduced by cooking. In addition, white beans have negligible amounts of PHA compared to colored beans. Phase 2 is a standardized white bean extract prepared using a specialized process which substantially inactivates haemagglutinating activity (HA) and trypsin inhibiting activity (TIA). The finished product contains less than 700 HA units per g and less than 20 TIA units per mg dry weight [31].

### Product equivalency

This review is focused on the development and clinical research of a proprietary product, Phase 2 Carb Controller (Pharmachem Laboratories, Kearny, NJ). We felt it was important to focus on this product as there is no evidence that carbohydrate blockers are equivalent. Early studies on the alpha-amylase inhibitor from white bean indicated that enzyme stabilization through specific manufacturing processes was key to an active product. The challenge of establishing biological equivalency for protein biopharmaceuticals has been highlighted in recent publications. The complexity of these molecules, and their production in living cells, makes the final product sensitive to changes in manufacturing conditions. Because of this, the European Medicine Agency has introduced a new regulatory pathway for biosimilars (also known as follow-on biologics) which mandates clinical trials in order to show therapeutic equivalence [49]. In the US, companies are expected to use the approval process for new branded drugs [50]. While the Phase 2 product is a dietary supplement, and not a pharmaceutical, we believe similar principals apply.

## Conclusions

Experiments conducted with the Phase 2 alpha-amylase inhibitor indicate that it reduces the rate of absorption of carbohydrates, thereby reducing the GI of foods. The evidence also indicates that Phase 2 promotes weight loss when taken concurrently with meals containing carbohydrates. The importance of reducing the GI of foods in weight management and type 2 diabetic control is indicated by an emerging body of evidence. Reducing the post-prandial spikes of glucose and insulin following a high GI meal may also reduce the risks of developing insulin resistance, which can lead to cardiovascular disease.

## Competing interests

Medicus Research has received research support grants from Pharmachem Laboratories. JKU has provided consulting services to Pharmachem Laboratories. MLB has provided consulting services to Medicus Research. The authors and Medicus Research do not endorse any brand or product.

## Authors' contributions

MLB and JKU were both involved in writing this review. They both read and approved the final manuscript.

## References

[B1] World Health Organization Obesity and overweight 2006 http://www.who.int/mediacentre/factsheets/fs311/en/index.html Ref Type: Online Source

[B2] Preuss HG Bean amylase inhibitor and other carbohydrate absorption blockers: effects on diabesity and general health J Am Coll Nutr 2009 28 266 276 20150600 10.1080/07315724.2009.10719781

[B3] Obiro WC Zhang T Jiang B The nutraceutical role of the Phaseolus vulgaris alpha-amylase inhibitor Br J Nutr 2008 100 1 12 10.1017/S0007114508879135 18331662

[B4] Food and Agriculture Organization, World Health Organization Carbohydrates in Human Nutrition: Report of a Joint FAO/WHO Report FAO Food and Nutrition paper 1998 66 1 140 9743703

[B5] Radulian G Rusu E Dragomir A Posea M Metabolic effects of low glycaemic index diets Nutr J 2009 8 5 10.1186/1475-2891-8-5 19178721 PMC2654909

[B6] Yamagishi S Matsui T Ueda S Fukami K Okuda S Clinical utility of acarbose, an alpha-glucosidase inhibitor in cardiometabolic disorders Curr Drug Metab 2009 10 159 163 10.2174/138920009787522133 19275550

[B7] Brand-Miller J McMillan-Price J Steinbeck K Caterson I Dietary glycemic index: health implications J Am Coll Nutr 2009 28 446S 449S 20234031 10.1080/07315724.2009.10718110

[B8] Barclay AW Petocz P McMillan-Price J Flood VM Prvan T Mitchell P Glycemic index, glycemic load, and chronic disease risk--a meta-analysis of observational studies Am J Clin Nutr 2008 87 627 637 18326601 10.1093/ajcn/87.3.627

[B9] Thomas DE Elliott EJ Baur L Low glycaemic index or low glycaemic load diets for overweight and obesity Cochrane Database Syst Rev 2007 CD005105 17636786 10.1002/14651858.CD005105.pub2 PMC9022192

[B10] Krishnan S Rosenberg L Singer M Hu FB Djousse L Cupples LA Glycemic index, glycemic load, and cereal fiber intake and risk of type 2 diabetes in US black women Arch Intern Med 2007 167 2304 2309 10.1001/archinte.167.21.2304 18039988

[B11] Villegas R Liu S Gao YT Yang G Li H Zheng W Prospective study of dietary carbohydrates, glycemic index, glycemic load, and incidence of type 2 diabetes mellitus in middle-aged Chinese women Arch Intern Med 2007 167 2310 2316 10.1001/archinte.167.21.2310 18039989

[B12] Schulze MB Liu S Rimm EB Manson JE Willett WC Hu FB Glycemic index, glycemic load, and dietary fiber intake and incidence of type 2 diabetes in younger and middle-aged women Am J Clin Nutr 2004 80 348 356 15277155 10.1093/ajcn/80.2.348

[B13] Schulze MB Schulz M Heidemann C Schienkiewitz A Hoffmann K Boeing H Fiber and magnesium intake and incidence of type 2 diabetes: a prospective study and meta-analysis Arch Intern Med 2007 167 956 965 10.1001/archinte.167.9.956 17502538

[B14] Englyst KN Englyst HN Carbohydrate bioavailability Br J Nutr 2005 94 1 11 10.1079/BJN20051457 16115326

[B15] Grabitske HA Slavin JL Low-digestible carbohydrates in practice J Am Diet Assoc 2008 108 1677 1681 10.1016/j.jada.2008.07.010 18926133

[B16] Higgins JA Resistant starch: metabolic effects and potential health benefits J AOAC Int 2004 87 761 768 15287677

[B17] Tundis R Loizzo MR Menichini F Natural products as alpha-amylase and alpha-glucosidase inhibitors and their hypoglycaemic potential in the treatment of diabetes: an update Mini Rev Med Chem 2010 10 315 331 10.2174/138955710791331007 20470247

[B18] McDougall GJ Stewart D The inhibitory effects of berry polyphenols on digestive enzymes Biofactors 2005 23 189 195 10.1002/biof.5520230403 16498205

[B19] Koh LW Wong LL Loo YY Kasapis S Huang D Evaluation of different teas against starch digestibility by mammalian glycosidases J Agric Food Chem 2010 58 148 154 10.1021/jf903011g 20050703

[B20] Moreno J Altabella T Chrispeels MJ Characterization of alpha-Amylase-Inhibitor, a Lectin-Like Protein in the Seeds of Phaseolus vulgaris Plant Physiol 1990 92 703 709 10.1104/pp.92.3.703 16667338 PMC1062357

[B21] Layer P Carlson GL DiMagno EP Partially purified white bean amylase inhibitor reduces starch digestion in vitro and inactivates intraduodenal amylase in humans Gastroenterology 1985 88 1895 1902 2581844 10.1016/0016-5085(85)90016-2

[B22] Layer P Zinsmeister AR DiMagno EP Effects of decreasing intraluminal amylase activity on starch digestion and postprandial gastrointestinal function in humans Gastroenterology 1986 91 41 48 2423408 10.1016/0016-5085(86)90436-1

[B23] Strocchi A Corazza GR Anania C Benati G Malservisi S Cherchi MV Quality control study of H2 breath testing for the diagnosis of carbohydrate malabsorption in Italy. The "Tenue Club" Group Ital J Gastroenterol Hepatol 1997 29 122 127 9646191

[B24] Strocchi A Corazza G Ellis CJ Gasbarrini G Levitt MD Detection of malabsorption of low doses of carbohydrate: accuracy of various breath H2 criteria Gastroenterology 1993 105 1404 1410 8224644 10.1016/0016-5085(93)90145-3

[B25] Brummer RJ Karibe M Stockbrugger RW Lactose malabsorption. Optimalization of investigational methods Scand J Gastroenterol Suppl 1993 200 65 69 10.3109/00365529309101578 8016574

[B26] Layer P Rizza RA Zinsmeister AR Carlson GL DiMagno EP Effect of a purified amylase inhibitor on carbohydrate tolerance in normal subjects and patients with diabetes mellitus Mayo Clin Proc 1986 61 442 447 2423813 10.1016/s0025-6196(12)61978-8

[B27] Brugge WR Rosenfeld MS Impairment of starch absorption by a potent amylase inhibitor Am J Gastroenterol 1987 82 718 722 2440298

[B28] Boivin M Zinsmeister AR Go VL DiMagno EP Effect of a purified amylase inhibitor on carbohydrate metabolism after a mixed meal in healthy humans Mayo Clin Proc 1987 62 249 255 2436011 10.1016/s0025-6196(12)61900-4

[B29] Boivin M Flourie B Rizza RA Go VL DiMagno EP Gastrointestinal and metabolic effects of amylase inhibition in diabetics Gastroenterology 1988 94 387 394 2446948 10.1016/0016-5085(88)90426-x

[B30] Jain NK Boivin M Zinsmeister AR Brown ML Malagelada JR DiMagno EP Effect of ileal perfusion of carbohydrates and amylase inhibitor on gastrointestinal hormones and emptying Gastroenterology 1989 96 377 387 2463204 10.1016/0016-5085(89)91561-8

[B31] Nicolosi R Hughes D Bechtel D Evaluation of the Generally Recognized as Safe (GRAS) status of Phase 2^® ^white bean (*Phaseolus vulgaris*) extract 2007 Bridgewater, NJ, Cantox Health Sciences International http://www.phase2info.com/pdf/science-dossier/phase2-gras-expert-report.pdf Ref Type: Online Source

[B32] Udani K The mighty bean European Baker 2005

[B33] Das Y Phase 2/Starch Lite 2007 Piscataway, NJ, ISSI Laboratories, Inc http://www.phase2info.com/pdf/Phase2_Study13.pdf ISSI no. P25036, 1-7. Ref Type: Online Source

[B34] Das Y Phase 2/Starch Lite in Chewing Gum 2007 Piscataway, NJ, ISSI Laboratories, Inc http://www.phase2info.com/pdf/Phase2_Study14.pdf ISSI no. P25036-B, 1-4. Ref Type: Online Source

[B35] Celleno L Tolaini MV D'Amore A Perricone NV Preuss HG A dietary supplement containing standardized Phaseolus vulgaris extract influences body composition of overweight men and women Int J Med Sci 2007 4 45 52 17299581 10.7150/ijms.4.45 PMC1796956

[B36] Rothacker D Reduction in body weight with a starch blocking diet aid: StarchAway comparison with placebo 2003 Leiner Health Products http://www.phase2info.com/pdf/Phase2_Study6.pdf Ref Type: Online Source

[B37] Wu X Xu X Shen J Perricone N Preuss H Enhanced weight loss from a dietary supplement containing standardized Phaseolus vulgaris extract in overweight men and women Journal of Applied Research 2010 10 73 79

[B38] Udani J Singh BB Blocking carbohydrate absorption and weight loss: a clinical trial using a proprietary fractionated white bean extract Altern Ther Health Med 2007 13 32 37 17658120

[B39] Thom E A randomized, double-blind, placebo-controlled trial of a new weight-reducing agent of natural origin J Int Med Res 2000 28 229 233 11092233 10.1177/147323000002800505

[B40] Erner S Meiss D Thera-Slim for Weight Loss: A randomized double-blind placebo controlled study 2003 http://www.phase2info.com/pdf/Phase2_Study8.pdf Ref Type: Online Source

[B41] Koike T Koizumi Y Tang L Takahara K Saitou Y The antiobesity effect and the safety of taking "Phaseolamin(TM) 1600 diet" J New Rem & Clin (Japanese) 2005 54 1 16

[B42] Osorio L Gamboa J Random multicenter evaluation to test the efficacy of Phaseolus vulgaris (Precarb) in obese and overweight individuals 2005 Internal Report http://www.phase2info.com/pdf/Phase2_Study10.pdf Ref Type: Online Source

[B43] Yamada J Yamamoto T Yamaguchi T Effects of combination of functional food materials on body weight, body fat percentage, serum triglyceride and blood glucose 2007 http://www.phase2info.com/pdf/Phase2_Study15.pdf Ref Type: Online Source

[B44] Udani J Hardy M Madsen DC Blocking carbohydrate absorption and weight loss: a clinical trial using Phase 2 brand proprietary fractionated white bean extract Altern Med Rev 2004 9 63 69 15005645

[B45] Vinson J Al Kharrat H Shuta D Investigation of an amylase inhibitor on human glucose absorption after starch consumption The Open Nutraceuticals Journal 2009 2 88 91 10.2174/1876396000902010088

[B46] Udani JK Singh BB Barrett ML Preuss HG Lowering the glycemic index of white bread using a white bean extract Nutr J 2009 8 52 10.1186/1475-2891-8-52 19860922 PMC2776021

[B47] Harikumar KB Jesil AM Sabu MC Kuttan R A preliminary assessment of the acute and subchronic toxicity profile of phase2: an alpha-amylase inhibitor Int J Toxicol 2005 24 95 102 10.1080/10915810590936364 16036768

[B48] Chokshi D Subchronic oral toxicity of a standardized white kidney bean (Phaseolus vulgaris) extract in rats Food Chem Toxicol 2007 45 32 40 10.1016/j.fct.2006.06.021 17045383

[B49] Schellekens H Biosimilar therapeutics-what do we need to consider? NDT Plus 2009 2 i27 i36 10.1093/ndtplus/sfn177 19461855 PMC2638545

[B50] Wiatr C US Biosimilar Pathway Unlikely to be Useddagger: Developers Will Opt for a Traditional BLA Filing BioDrugs 2011 25 63 67 10.2165/11588410-000000000-00000 21222498

